# The genus *Parasola* in Pakistan with the description of two new species

**DOI:** 10.3897/mycokeys.30.21430

**Published:** 2018-02-27

**Authors:** Shah Hussain, Habib Ahmad, Sadiq Ullah, Najam-Ul-Sehar Afshan, Donald H. Pfister, Hassan Sher, Haidar Ali, Abdul N. Khalid

**Affiliations:** 1 Center for Plant Sciences and Biodiversity, University of Swat, Swat, Pakistan; 2 Department of Botany, Hazara University, Mansehra 21300, Pakistan; 3 Islamia College, Peshawar, Pakistan; 4 Centre for Undergraduate Studies, University of the Punjab, Lahore 54590, Pakistan; 5 Department of Organismic and Evolutionary Biology, Harvard University, Cambridge Massachusetts 02138, USA; 6 Department of Botany, University of the Punjab, Lahore 54590, Pakistan

**Keywords:** Basidiomycota, diversity, *Parasola*, phylogeny, taxonomy

## Abstract

*Parasola* is a genus of small, veil-less coprinoid mushrooms in the family Psathyrellaceae (Agaricales). The genus is not well documented in Asia, specifically in Pakistan. In this study we describe two new species *Parasola glabra* and *P. pseudolactea* from Pakistan, based on morphological and molecular data. Phylogeny based on three DNA regions: nuc rDNA region encompassing the internal transcribed spacers 1 and 2 along with the 5.8S rDNA (ITS), nuc 28S rDNA D1-D2 domains (28S) and translation elongation factor 1*α* gene (*TEF1α*) show that the new taxa are clustered in a clade formed by the members of section Parasola of genus *Parasola*. *Parasola glabra* with grayish pileus, slightly depressed pileal disc, lamellae separated from the stipe by pseudocollarium, basidiospores 14.5–16.5 × 9.5–11.5 × 8.0–10.5 µm, in front view broadly ovoid to oblong, some with rhomboidal outline, in side view ellipsoid, with eccentric germ-pore of 1.5 µm diameter. *Parasola pseudolactea* with yellowish brown to dull brown pileus, disc indistinctly umbonate, lamellae free, pseudocollarium absent, basidiospores 13.5–14.5 × 10.5–12.0 × 9.5–10.5 µm, in face view rounded triangular to heart shaped, rarely ovoid to subglobose, in side view ellipsoid to oblong, with eccentric germ-pore of 1.5 µm diam. In addition to these new species, *P. auricoma* and *P. lilatincta* were also studied. Morphological descriptions for the new species and comparison with known *Parasola* species are provided. Our observations highlight the diversity of *Parasola* in northern Pakistan and further document the need for additional systematic focus on the region’s fungi.

## Introduction

*Parasola* Redhead, Vilgalys & Hopple is a genus of small, veil-less coprinoid mushrooms belonging to family Psathyrellaceae Vilgalys, Moncalvo & Redhead (Redhead et al. 2001, Nagy et al. 2009, Schafer 2010). These fungi are saprotrophs of decayed organic matter in bare soil, grassland, on woody debris including wood chips and on herbivore dung (Schafer 2014). The genus *Parasola* typified by *Parasola plicatilis* (Curtis) Redhead, Vilgalys & Hopple (Redhead et al. 2001), currently comprises 18 established species, distributed world-wide. The genus is well documented in Europe (Orton and Watling 1979, Uljé and Bas 1988, Uljé and Bender 1997, Schafer 2014, Szarkándi et al. in press). Some species are reported from North America, Africa, Lesser Antilles (Pegler 1966, 1983, Dennis 1970), and Asia (Ahmad 1980, Pegler 1986, Hongo 1987, Hussain et al. 2016, 2017) and Australia (Grgurinovic 1997).

Species of Parasola are divided into section Auricomi (Singer) D.J. Schaf. and section Parasola Redhead, Vilgalys & Hopple (previous references to Parasola section Glabri (Lange) D.J. Schaf. – see Schafer (2010) – should be replaced by Parasola section Parasola to conform with the International Code of Nomenclature for Algae, Fungi and Plants (Schafer, D.J., personal communication). The sections are distinguished on the basis of presence or absence of hair-like, golden- to dark brown, thick walled sclerocystidia in the pileipellis (Schafer 2010). In mature fruitbodies during basidiospore discharge, the gill cystidia of *Parasola* lose turgor and collapse, a characteristic feature of the genus (Nagy et al. 2009).

Basidiospore shape and size are the main descriptive features for species identification in *Parasola* (Nagy et al. 2009, 2010, Schafer 2014, Hussain et al. 2017).

Previously, five species of this genus (*Parasola auricoma* (Pat.) Redhead, Vilgalys & Hopple, *P. lilatincta* (Bender & Uljé) Redhead, Vilgalys & Hopple, *P. malakandensis* S. Hussain, N. Afshan & H. Ahmad, *P. plicatilis* and *P. setulosa* (Berk. & Broome) Redhead, Vilgalys & Hopple) have been reported from Pakistan (Ahmad 1980, Hussain et al. 2016, 2017). In this study, we describe two new species *P. glabra* and *P. pseudolactea*, based on morphological characters and phylogenetic analyses of nuc rDNA region encompassing the internal transcribed spacers 1 and 2, along with the 5.8S rDNA (ITS), nuc 28S rDNA D1-D2 domains (28S) and translation elongation factor 1α gene (*TEF1α*). In addition to these new species we also studied *P. auricoma* and *P. lilatincta.*

## Materials and methods

### Sampling and morphological characterization

Specimens were collected from Malakand, Shangla and Swat districts of Khyber Pakhtunkhwa, Pakistan in summer seasons, 2013–2017. Basidiomata were photographed, tagged and field notes were made. Munsell (1975) was used for determination of color. The specimens were air-dried and kept in zip-lock bags. Specimens examined in this study are deposited in the Herbaria of Hazara University Mansehra, Pakistan (HUP), University of the Punjab, Lahore, Pakistan (LAH) and University of Swat, Pakistan (SWAT).

For anatomical studies slides were prepared in 5% aqueous KOH (w/v). Microscopic features such as size and shape of basidiospores, basidia, cheilocystidia, pleurocystidia and pileipellis were studied under a light microscope (MX4300H, Meiji Techo Co., Ltd., Japan) with at least 20 structures measured in each instance. Cheilocystidia and pleurocystidia were observed and measured by cutting the gill edge from the rest of gill to avoid confusion between the two types of cystidia. In the case of basidiospores, 50 spores were measured in face view and/or side view through 1000× magnification with a calibrated optical micrometer and measurements were rounded to the nearest 0.5 µm. Basidiospores measurements are presented as follows: length range × breadth range × width range. Q values were calculated as: Q_1_ = length divided by breadth; Q_2_ = length divided by width (Nagy et al. 2010).

### DNA extraction, PCR and sequencing

We extracted genomic DNA using the DNeasy Plant Mini Kit (Qiagen, Redwood City, California, USA.). We amplified nuc rDNA internal transcribed spacer (ITS) and 28S loci and translation elongation factor 1α gene (*TEF1α*) using the primer combinations ITS1F/ITS4; LR0R/LR5 and EF1-983F/EF1-1567R, respectively (White et al. 1990, Gardes and Bruns 1993, Rehner and Buckley 2005). For PCR amplification, we followed Hussain et al. (2017). PCR products were purified using the QIAquick PCR Purification kit (Qiagen). Sequencing was performed with the same PCR primers using the Big Dye Sequencing Kit v.3.1 on an ABI-3730-XL DNA Analyzer (Applied Biosystems, Foster City, California, USA). Sequences produced for this study have been deposited in GenBank (Table 1).

**Table 1.** Voucher numbers, geographic origins and GenBank Accession numbers for the specimens included, in boldface are sequences produced in this study.

Species	Geographic origin	Voucher number	GenBank Accessions		
			ITS	28S	*TEF1*α
***Parasola auricoma***	**Pakistan**	**LAH-SHP-P6**	KX212106	**KY461729**	**MG587083**
	**Pakistan**	**LAH-SHP-P7**	**KY461721**	**KY461730**	**MG587084**
	**Pakistan**	**LAH-SHP-P11**	**KY621802**	**KY461728**	
	Hungary	NL0268	FM163186	FM160723	
	Hungary	NL0087	FM163185	FM160724	FM897236
*P. conopilus*	Hungary	NL0465	FM160686	FM163223	
	Hungary	NL0286	FM160685	FM163224	
	Hungary	NL0285	FM160684	FM163225	KJ732832
***P. glabra***	**Pakistan**	**LAH-SHP-5** (Holotype)	**KY461717**	**KY621806**	**KY461735**
	**Pakistan**	**HUP-SHP-23**	**KY461718**	**KY621805**	
*P. hercules*	Netherlands	Uljé 1269 (L)	FM163190	FM160719	
	Netherlands	L146 holotype	HQ847027	HQ847112	
*P. kuehneri*	Netherlands	Uljé 904 (L)	FM163191	FM160718	
*P. lactea*	Hungary	NL0466	FM163192	FM160717	FM897241
	Sweden	NL0095	FM163188	FM160721	
	Germany	NL0283	FM163194	FM160715	FM897239
	Sweden	NL0288	FM163193	FM160716	
	Hungary	NL6601	FM163187	FM160722	
	USA	MICH232885	KM403384		
	Latvia	KuP6.2.2.1	KP698198		
***P. pseudolactea***	**Pakistan**	**HUP-SU-412** (Holotype)	**KY461719**	**KY621799**	**KY461733**
	**Pakistan**	**HUP-SU-413**	**KY461720**	**KY621800**	**KY461734**
***P. lilatincta***	**Pakistan**	**LAH-SHP-8**	**KY461722**	**KY461725**	**KY461731**
	**Pakistan**	**LAH-SHP-31**	**KY461723**	**KY461726**	**KY461732**
	**Pakistan**	**LAH-SHP-12**	**KY461724**	**KY461727**	
	Hungary	NL0683	FM163203	FM160706	FM897231
	Hungary	NL0660	FM163195	FM160714	FM897230
	Hungary	NL0472	FM163199	FM160709	
	Hungary	NL0667	FM163198	JQ045886	
	Pakistan	SH4	KP886462		
	Pakistan	SHP2	KP886463		
	Pakistan	SHP9	KP886464		
P. aff. lilatincta	Hungary	NL0086	FM163204	FM160705	
	Sweden	NL0096	FM163205	FM160704	
*P. megasperma*	Denmark	C 19683	FM163206	FM160703	
	Sweden	NL1924	FM163208	FM160701	FM897232
*P. malakandensis*	Pakistan	LAH-SHP-17	KU599827	KU599830	KU599832
	Pakistan	HUP 17501	KP738713	KU599829	KU599831
*P. misera*	Hungary	NL0677	FM160698	FM163211	FM897240
	Hungary	NL0280	FM160699	FM163210	
	Hungary	NL0490	FM163209	FM160700	
*P. plicatilis*	Sweden	NL0477	FM163212	FM160697	FM897235
	Hungary	NL0295	FM163216	FM160693	FM897242
*P. plicatilis*	Sweden	NL0097	FM163215	FM160694	
	Hungary	NL0075	FM163214	FM160695	
	Hungary	NL0284	FM163189	FM160720	
*P. schroeteri*	Netherlands	LBrier:1051999	FM163219	FM160690	
*P. setulosa*	Hungary	L32	HQ847030	HQ847115	
*Parasola* sp.	Norway	NL3167	JN943136	JQ045865	
*Parasola* sp.	Norway	NL3621	JN943134	JQ045875	
*Parasola* sp.	Hungary	NL4175	HQ847025	HQ847110	
*Parasola* sp.	Hungary	NL0287	FM163218	FM160691	
*Parasola* sp.	Hungary	NL2952	HQ847028		
*Psathyrella candolleana*	Hungary	NL2937	FN396114	FN396165	FN396220

### Alignments and phylogenetic inference

ITS, 28S and *TEF1α* sequences were aligned using BIOEDIT v 7.2.5 (Hall 1999) and CLUSTAL X 2.1 (Larkin et al. 2007). The ITS, 28S and *TEF1α* alignments were concatenated into a supermatrix. *Psathyrella candolleana* (Fr.) Maire was selected as outgroup. Alignments are submitted to TreeBase (Treebase ID 21639). Phylogenetic inference was conducted using Bayesian and Maximum Likelihood (ML) methods. For Bayesian inference, we used BEAST 1.6.2 (Drummond and Rambaut 2007) with a Markov chain Monte Carlo (MCMC) coalescent approach. A Yule tree prior (Gernhard 2008) was used in all simulations, and the starting tree was randomly generated. Four independent runs were undertaken. Chain length was 10 million generations, with a sampling frequency of 1000. TRACER 1.6 (Rambaut et al. 2014) was used to check the effective sample size (ESS), and burn in values were adjusted to achieve an overall ESS (Effective Sample Size) of ≥ 200. Maximum clade credibility tree (20% burn-in) was generated using TREEANNOTATOR 1.6.2 (Drummond and Rambaut 2007). Maximum Likelihood analyses were run in RAXML-VI-HPC (Stamatakis 2006). Rapid bootstrap analysis/search for best-scoring ML tree (-f a) was configured. For the bootstrapping phase, the GTRCAT model was selected. One thousand rapid bootstrap replicates were run. Nodes were considered strongly supported when maximum likelihood bootstrap (MLB) were ≥ 70% and Bayesian posterior probability (BPP) were ≥ 0.95.

## Results

### Phylogenetic analyses

Sequence length varied from 631 bp (SHP-8) to 644 bp (SHP-11) for our 10 new ITS (ITS1-5.8S-ITS2) sequences and 1042 bp (SHP-12) to 1144 bp (SHP-8) for 10 28S sequences. The 7 *TEF1a* sequences generated for this study varied from 402 bp (SHP-5) to 502 bp (SU-412). The ITS dataset contained 52 taxa and 631 characters long after being trimmed (Trimming was done manually in BIOEDIT v 7.2.5). The combined ITS-28S dataset represented 47 taxa and 1892 characters long after being trimmed. Similarly, the combined ITS-28S-*TEF1a* dataset comprised 20 species and with 2890 nucleotides, after being trimmed.

**Figure 1.**
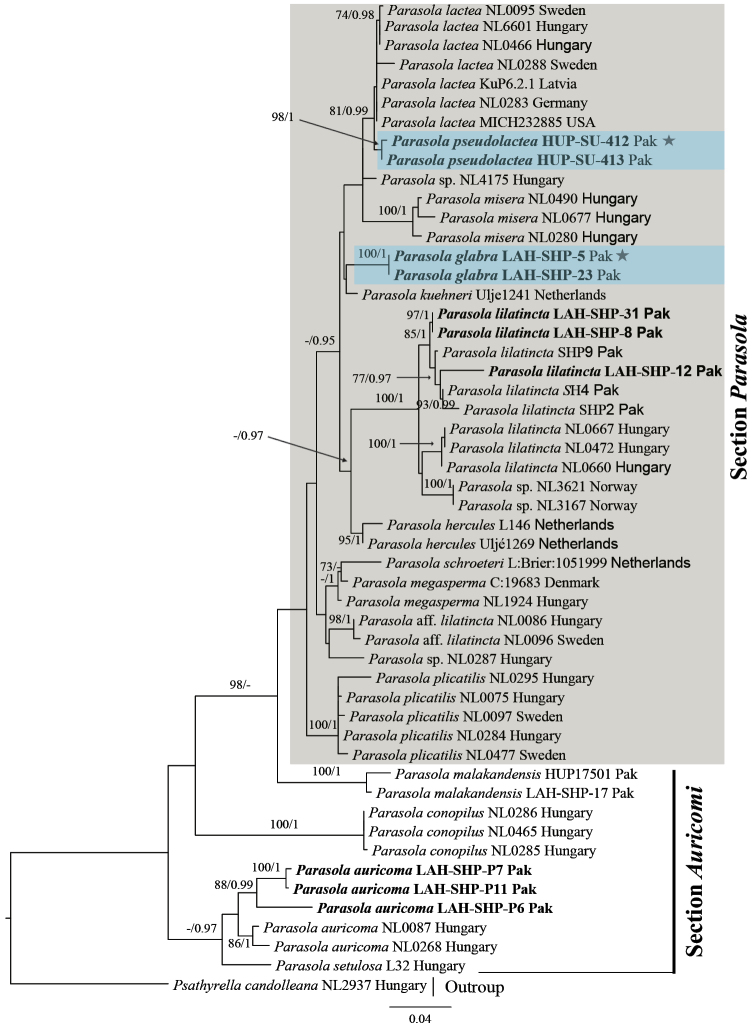
Phylogeny of *Parasola* species based on 52 ITS sequences. Our sequences are indicated in boldface. Other sequences are from Nagy et al. (2009). Numbers above or below branches indicate maximum likelihood bootstrap percentages followed by Bayesian posterior probabilities. Species in section Parasola are gray highlighted where the new species are shown as light-blue highlighted, while the HOLOTYPE collection for *P. glabra* (LAH-SHP-5) and *P. pseudolactea* (HUP-SU-412) are represented by stars (*).

**Figure 2.**
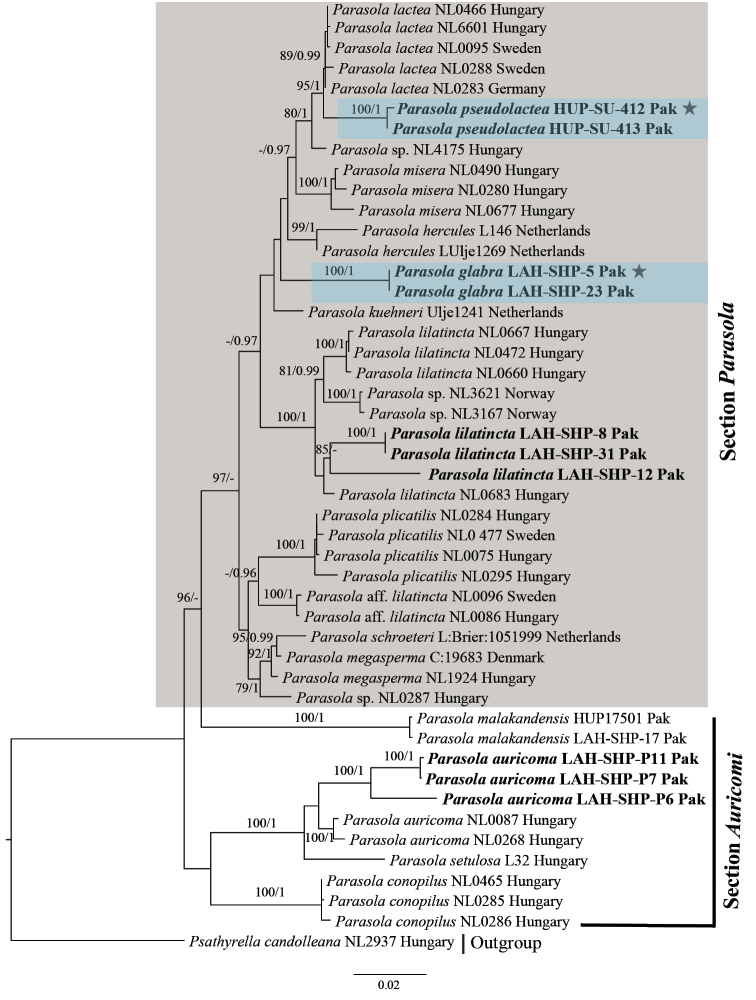
Phylogeny of *Parasola* species based on 47 sequences of combined ITS-28S dataset. Our sequences are indicated in boldface. Other sequences are from Nagy et al. (2009). Numbers above or below branches indicate maximum likelihood bootstrap percentages followed by Bayesian posterior probabilities. Species in section Parasola are gray highlighted where the new species are shown as light-blue, while the HOLOTYPE collection for *P. glabra* (LAH-SHP-5) and *P. pseudolactea* (HUP-SU-412) are represented by stars (*).

**Figure 3.**
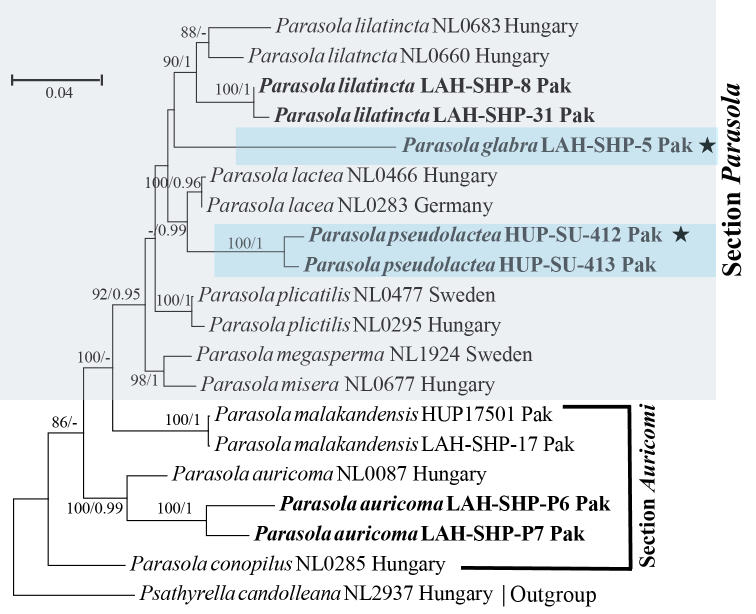
Phylogeny of *Parasola* species based on 20 sequences of combined ITS-28S-*TEF1α* dataset. Our sequences are indicated in boldface. Other sequences are from Nagy et al. (2009, 2011). Numbers above or below branches indicate maximum likelihood bootstrap percentages followed by Bayesian posterior probabilities. Species in section Parasola are light-brown highlighted where the new species are shown as light-blue, while the HOLOTYPE collection for *P. glabra* (LAH-SHP-5) and *P. pseudolactea* (HUP-SU-412) are represented by stars (*).

The results of phylogenetic analyses of ITS, ITS-28S and combined ITS-28S-*TEF1a* datasets are summarized in Figures 1, 2 and 3, respectively. Each tree represents ML phylogeny produced by RAXML analysis. Maximum likelihood bootstrap (MLB) percentages > 70% are given above or below the branch node, followed by Bayesian posterior probabilities (BPP) > 0.95. The novel sequences in this study are represented in boldface (Figures 1, 2 and 3), their Genbank accessions are provided in Table 1.

Using Bayesian and ML methods, *P. auricoma*, *P. conopilus*, *P. setulosa* and *P. malakandensis* were recovered as basal groups with strong support, collectively forming section Auricomi, whereas species of section Parasola fall in a single clade represented as gray highlighted, called ‘the crown *Parasola*’ clade (Nagy et al. 2009). Statistical support for the specimens that represent *P. pseudolactea* was strong in ITS dataset (MLB 98% and BPP 1), and excellent in combined ITS-28S and ITS-28S-*TEF1a* datasets, respectively (MLB 100% and BPP 1). Similarly, statistical support for *P. glabra* in both ITS and combined ITS-28S datasets was maximal (MLB 100% and BPP 1). In combined ITS-28S-*TEF1a* dataset *P. glabra* was represented by a single specimen and poorly recovered (Figure 3).

## Taxonomy

### Parasola glabra

Taxon classificationFungiAgaricalesPsathyrellaceae

Hussain, Afshan, Ahmad & Khalid sp. nov.

MB819601

Figures 4 , 5

#### Diagnosis.

The diagnostic features of *Parasola glabra* are grayish pileus, deeply plicate towards margin; disc slightly depressed, strong reddish orange; lamellae free, separated from the stipe by pseudocollarium; basidiospores 14.5–16.5 × 9.5–11.5 × 8.0–10.5 µm, in front view broadly ovoid to oblong, some with rhomboidal outline, in side view ellipsoid, with eccentric germ-pore of 1.5 µm diam.

#### Type.

PAKISTAN. Khyber Pakhtunkhwa Province, Malakand, Qaldara, scattered under herbaceous plants, 480 m alt., 15 August 2014, S. Hussain SHP5 (holotype: LAH SH-P5; GenBank accessions: ITS = KY461717; 28S = KY621806; *TEF1α* = KY461735).

#### Description.

Pileus 20–30 mm diam, initially subglobose, later convex to hemispheric; at first smooth, without veil, the center glabrous at maturity, becoming deeply plicate towards the margin; light gray (2.5R 6/2) to moderate gray (7.5R 6/2); disc slightly depressed, strong reddish orange (7.5R 5/12). Lamellae free, fairly crowded, separated from the stipe by pseudocollarium, 0–2 lamellulae, regular, initially whitish, then dark brown becoming black at maturity, finally losing turgor and collapsing. Stipe 30–60 × 2–3 mm, central, equal, smooth, slightly sub-bulbous at the base, hollow, white, fragile, without annulus.

**Figure 4.**
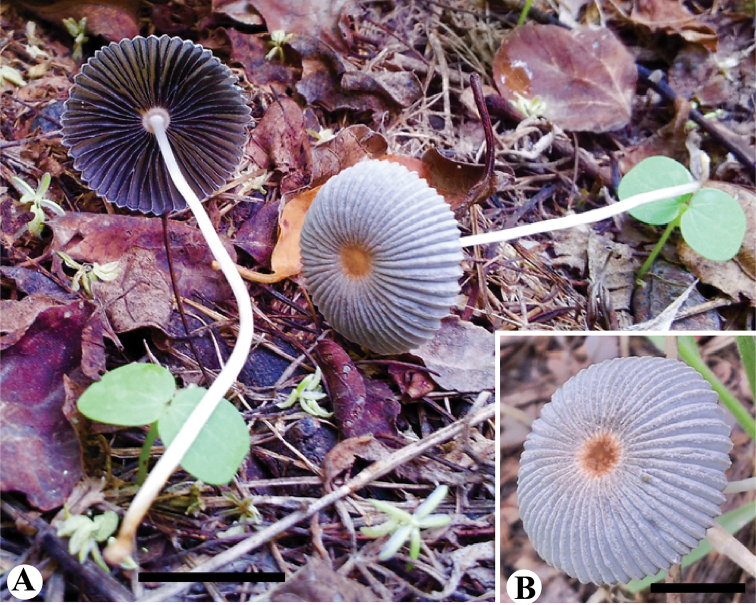
Basidiomata of *Parasola glabra* sp. nov. **A, B** Collection SHP-5 (HOLOTYPE LAH-SHP-5). Scale bars: 20 mm.

**Figure 5.**
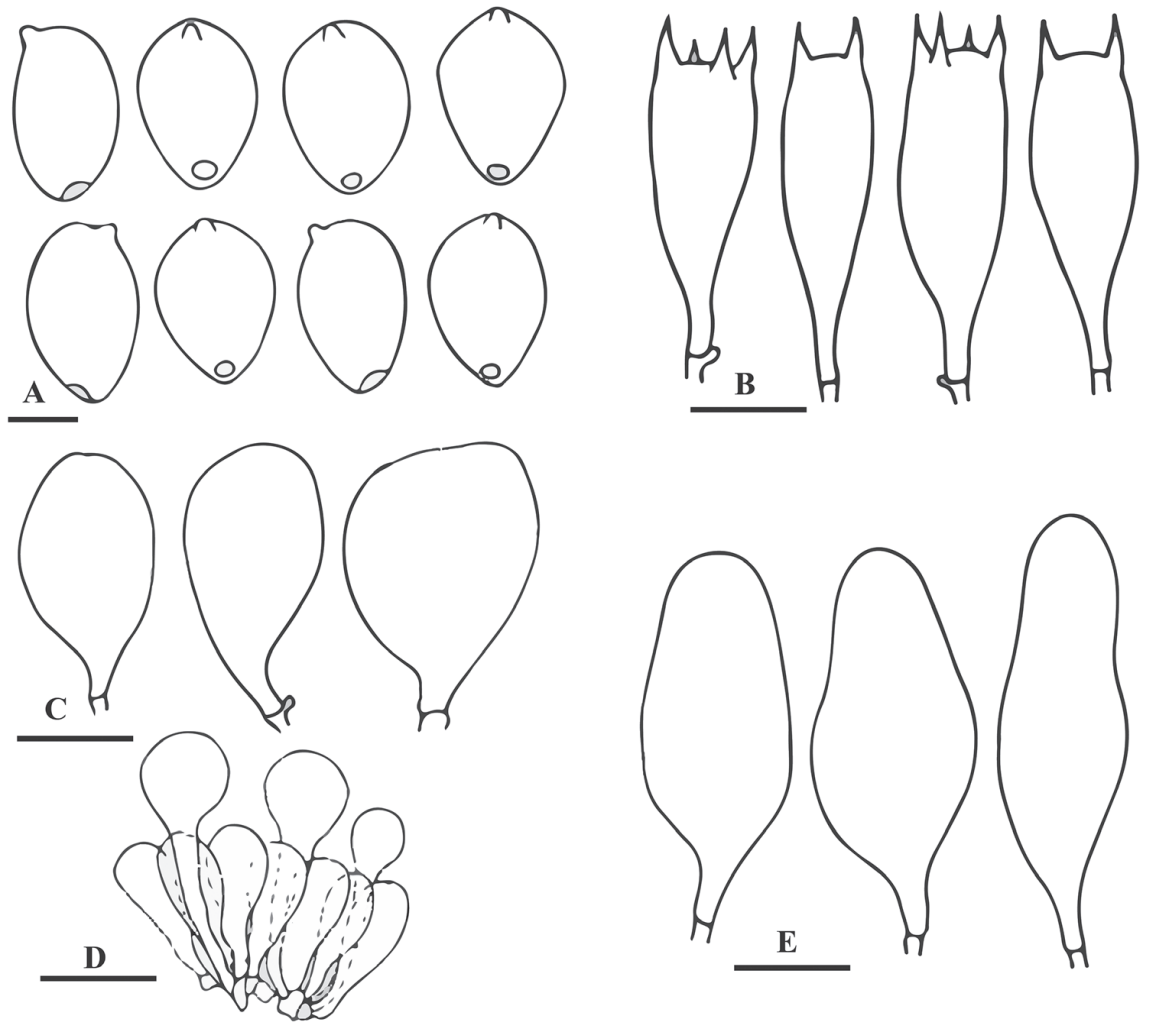
Anatomical features of *Parasola glabra* sp. nov. (LAH-SHP-5). **A** Basidiospres **B** Basidia **C** Pleurocystidia **D** Pileipellis **E** Cheilocystidia. Scale bars: 12 µm (**A**), 20 µm (**B–E**).

Basidiospores (13)14.5–16.5(18) × (7.5)9.5–11.5(15) × (9)8.0–10.5(11.5) µm, on average 15.8 × 10.9 × 10.1 µm, Q_1_ = 1.3–1.5, Q_2_ = 1.4–1.6, avQ = 1.4; in face view broadly ovoid to oblong, some with rhomboidal outline, in side view ellipsoid, germ-pore eccentric and upto 1.5 µm diam; wall upto 1.5 µm thick, dark brown to blackish in KOH. Basidia 28–41 × 10–13 µm, clavate to cylindrical, 4-spored, hyaline in KOH. Cheilocystidia 50–63 × 17–23 µm, oblong, ellipsoid, narrowly to broadly utriform, hyaline. Pleurocystidia 60–75 × 22–38 µm, clavate to broadly lageniform, hyaline. Pileipellis hymeniform, consisting of clavate cells 47–60 × 13–16 µm, bright yellow at the base in KOH. Clamp connections present mostly in the pileipellis and at the base of basidia. Sclerocystidia absent.

#### Habitat and distribution.

Saprotrophic, scattered under herbaceous plants on grass land. So far only known from the lowland of northern Pakistan. This species is, however, common in lowland northwest Pakistan.

#### Etymology.

Specific epithet ‘*glabra*’ refers to the glabrous cap found in species of section Parasola of the genus *Parasola*, where this species belongs.

#### Additional specimen examined.

PAKISTAN, Khyber Pakhtunkhwa Province, Malakand, Qaldara, 480 m alt., 28 May 2015, S. Hussain SHP23 (HUP SHP-23).

#### Comments.

The distinguishing features of the new species *P. glabra* are: basidiospores broadly ovoid to oblong, some with rhomboidal outline in face view, ellipsoid in side view, on range 14.5–16.5 × 9.5–11.5 × 8.0–10.5 µm, pileus light gray to moderate gray but reddish orange at the disk, without sclerocystidia. Lacking sclerocystidia, *P. glabra* belongs in section Parasola. On basidiospore dimensions, it could be thought close to *P. plicatilis* and *P. megasperma* (P.D. Orton) Redhead, Vilgalys & Hopple but these are distinguishable on the basis of spores shape, length and breadth together and on the color of the cap disk. Using maximum likelihood phylogeny, these two species are clearly distinct from *P. glabra* and, based on ITS and 28S loci, the more closely related species are: *P. hercules* (Uljé & Bas) Redhead, Vilgalys & Hopple; *P. kuehneri* (Uljé & Bas) Redhead, Vilgalys & Hopple; *P. lilatincta* and *P. schroeteri* (P. Karst.) Redhead, Vilgalys & Hopple. The new species can be distinguished from these species on account of basidiospore morphology: among these species, *P. hercules* has the largest spore breadth (11.3–16.9 µm), followed by *P. schroeteri* (9–13 µm), *P. glabra* (9.5–11.5 µm), *P. lilatincta* (9–11.2 µm) and smallest spore breadth (5.5–8.4 µm) in *P. kuehneri*. On the basis of basidiospore length/breadth ratio (Q_1_), the new taxon *P. glabra* (Q_1_ = 1.3–1.5), can be easily distinguished from these species: in *P. hercules* (Q_1_ = 1.04–1.28), *P. schroeteri* (Q_1_ = 1.16–1.27), *P. lilatincta* (Q1 = 1.14–1.33) and *P. kuehneri* (Q1 = 1.12–1.28), respectively (Nagy et al. 2010, Schafer 2014). Comparison of morphological characters of *P. glabra* with regards to these and other species of section Parasola genus *Parasola* are set out further in Table 2.

**Table 2.** Characteristics distinguishing *Parasola glabra* and *P. pseudolactea* from the remaining species in section Parasola.

Taxa	Pileus diam; and pileus color	Stipe size	Basidiospores size, length/breadth (Q_1_), length/width (Q_2_) ratios	Basidiopores shape and germ-pore position	References
***P. glabra***	20–30 mm diam, light-gray to moderate-gray	30–60 × 2–3 mm	15.8 × 10.9 × 10.1 µm; Q_1_ = 1.3–1.5, Q_2_ = 1.4–1.6, avQ = 1.4	In face view broadly ovoid to oblong, some with rhomboidal outline, in side view ellipsoid; germ-pore eccentric, upto 1.5 µm diam.	Observed during this study.
*P. hercules*	15–20 mm diam, orange-brown to red-brown	75 × 1.5 mm	15.83 × 15.42 × 10.63 µm;Q_1_ = 1–1.15, Q_2_ = 1.4–1.5	In face view rounded triangular to quadrangular, rarely subglobose to ovoid, in side view ellipsoid to amygdaliform; germ-pore eccentric, upto 2.7µm diam.	Nagy et al. 2010, Schafer 2014.
*P. kuehneri*	35 mm diam, dark light grayish-brown	100 × 3 mm	9.36 × 7.85 × 5.9 µm;Q_1_ = 1.1–1.2, Q_2_ = 1.4–1.6	In face view ovoid to rounded triangular, rhomboid to mitriform, in side view amygdaliform; germ- pore eccentric, 1.5 µm diam.	Nagy et al. 2010, Schafer 2014.
*P. lactea*	15–23 mm diam, yellow-brown to dull red-brown	140 × 3 mm	10.73 × 8.81 × 6.73 μm; Q_1_ = 1.02–1.25, Q_2_ = 1.66–2.10	In face view mostly broadly ovoid to subglobose, rarely angular to rounded triangular, in side view broadly ellipsoid to ellipsoid; germ-pore eccentric, upto 1.8 μm diam.	Nagy et al. 2010, Schafer 2014.
***P. pseudolactea***	15–25 mm diam, initially yellow-brown to dull-brown, moderate gray at maturity	30–50 × 1 mm	14.0 × 11.3 × 9.7 µm; Q_1_ = 1.3–1.5, Q_2_ = 1.4–1.5, avQ = 1.4	In face view mostly rounded triangular to heart shape, rarely ovoid to subglobose, in side view ellipsoid to oblong, germ-pore eccentric, upto 1.5 µm diam.	Observed during this study
*P. lilatincta*	30–50 mm diam, dark reddish brown, not plicate	70–100 ×2–4 mm	14.4 × 10.8 × 9.2 µm; Q_1_ = 1.3–1.4, Q_2_ = 1.3–1.5	In face view rounded triangular to quadrangular, in side view ellipsoid to amygdaliform; germ-pore eccentric, upto 2.5 µm diam.	Uljé and Bender 1997, Nagy et al. 2010, Schafer 2014, Hussain et al. 2016.
*P. megasperma*	35 mm diam, chestnut-brown to red-brown or ochre-tawny	50–100 × 1.5–3 mm	16.5 × 10.66 × 8.5 μm; Q_1_ = 1.40–1.78, Q_2_ = 1.83–1.95	In face view ellipsoid to broadly ellipsoid, rarely ovoid, in side view ellipsoid to subamygdaliform; germ-pore slightly eccentric, upto 2.3 µm diam.	Nagy et al. 2010, Schafer 2014.
*P. misera*	2–5 × 1–3 mm, tawny-orange to cinnamon-brown	50 × 0.5 mm	7.0–10.6 × 6.5–10.0 × 5.9–6.6 μm	In face view heart-shape to rounded triangular, irregularly globose, in side view ellipsoid; sometimes broader than long; germ-pore eccentric.	Schafer 2014.
*P. plicatilis*	35 mm diam, yellow-brown to dull pinkish-brown	30–70 × 0.5–3 mm	12.41 × 8.21 × 7.14 μm; Q_1_ = 1.34–1.67, Q_2_ = 1.61–1.86	In face view mostly leminiform-subhexagonal, rarely ovoid, in side view ellipsoid to subamygdaliform; germ-pore eccentric, 2.3 µm diam.	Nagy et al. 2010, Schafer 2014.
*P. schroeteri*	20–30 mm diam, yellow-brown to grayish red-brown	40–60 × 1 mm	14.44 × 11.83 × 9.72 μm, Q_1_ = 1.16–1.27, Q_2_ = 1.46–1.68	In the face view rounded triangular to subglobose, in side view ovoid to amygdaliform; germ-pore eccentric, upto 2.5 μm diam.	Uljé and Bender 1997, Nagy et al. 2010, Schafer 2014.

### Parasola pseudolactea

Taxon classificationFungiAgaricalesPsathyrellaceae

Sadiqullah, Hussain & Khalid sp. nov.

MB819600

Figures 6 , 7

#### Diagnosis.

Pileus yellowish brown to dull brown, deeply plicate towards margin; disc subumbilicate, deep orange yellow; lamellae free, pseudocollarium absent; basidiospores 13.5–14.5 × 10.5–12.0 × 9.5–10.5 µm, in face view rounded triangular to heart shape, rarely ovoid to subglobose, in side view ellipsoid to oblong, with eccentric germ-pore of 1.5 µm diam; sclerocystidia absent.

#### Type.

PAKISTAN, Khyber Pakhtunkhwa Province, Shangla, solitary to scattered under *Quercus incana*, 1480 m alt., 9 July 2014, Sadiq Ullah SU412 (holotype: HUP SU-412; GenBank accessions: ITS = KY461719; 28S = KY621799; *TEF1α* = KY461733).

#### Description.

Pileus 15–25 mm diam, initially obtusely conical, later becoming applanate and deeply plicate towards margin; yellowish brown to dull brown (10YR 6/4) when young, moderate gray (7.5R 6/2) on maturity; disk subumbilicate, deep orange-yellow (7.5YR 6/12). Lamellae free, 0–2 lamellulae, distant, pseudocollarium absent, initially dark gray, becoming blackish at maturity and finally losing turgor and collapsing. Stipe 30–50 × 1 mm, equal, smooth, grayish-brown, translucent, hollow, without annulus.

**Figure 6.**
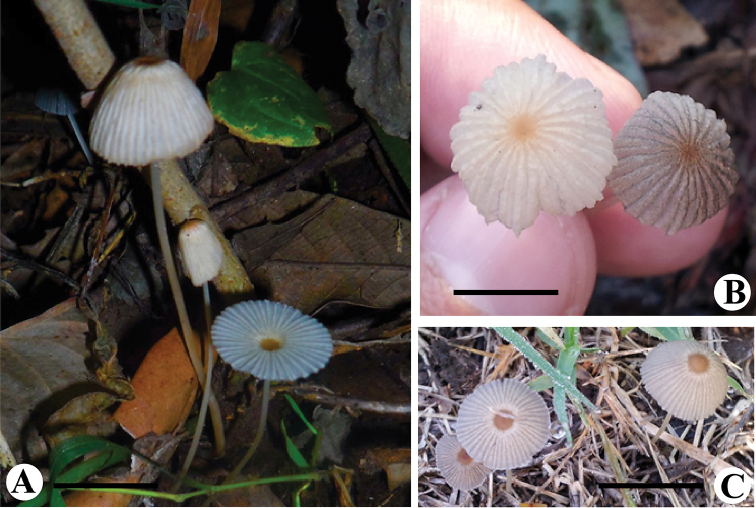
Basidiomata of *Parasola pseudolactea* sp. nov. *P. auricoma* and *P. lilatincta*. **A** *Parasola pseudolactea* sp. nov. collection SU-412 (HOLOTYPE HUP SU-412) **B** *Parasola lilatincta* collection SHP-8 (HUP-SHP-8) **C** *Parasola auricoma* collection SHP7 (LAH-SHP-7). Scale bars: 20 mm.

**Figure 7.**
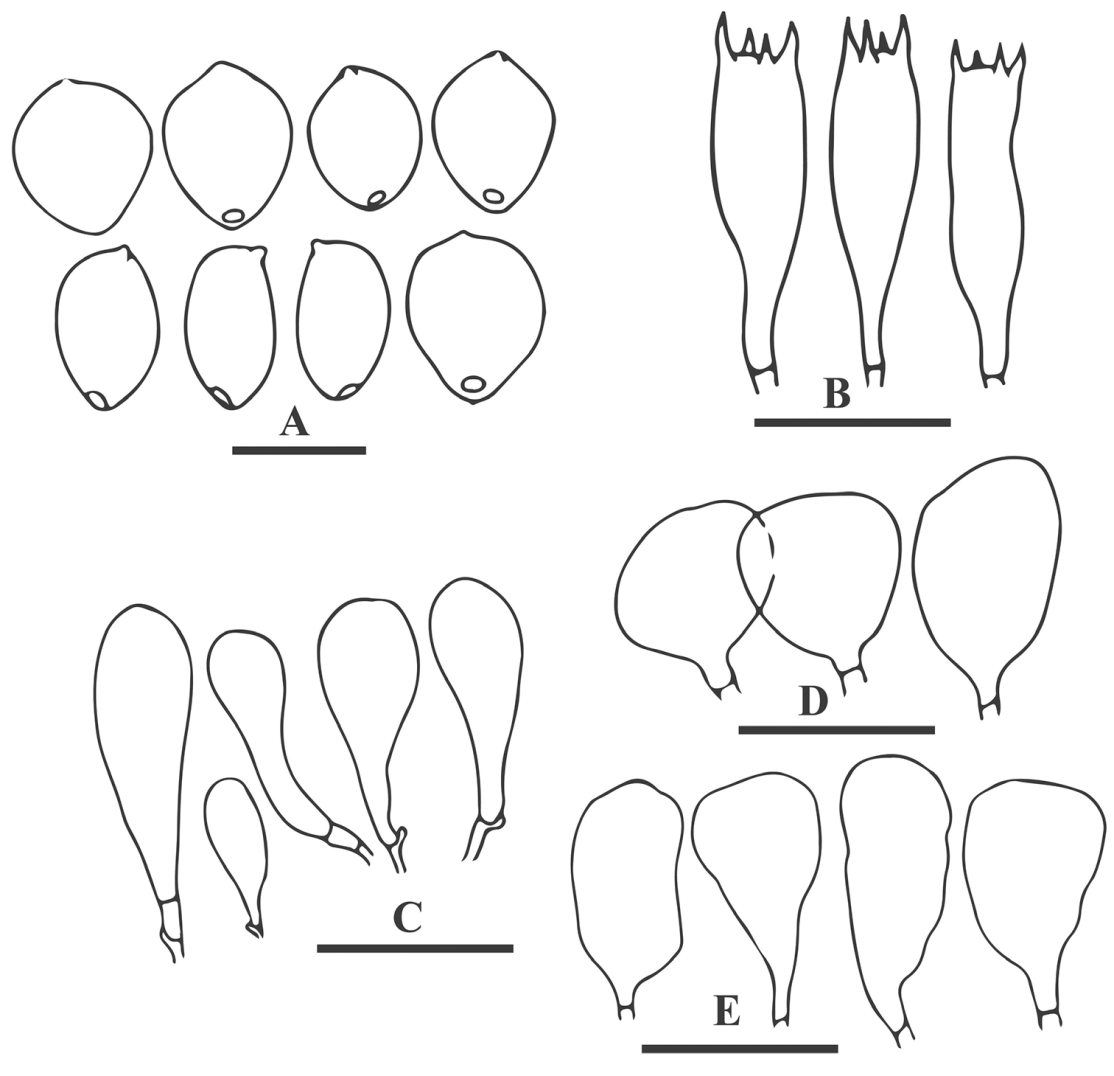
Anatomical features of *Parasola pseudolactea* sp. nov (HUP-SU-412). **A** Basidiospores **B** Basidia **C** Pileipellis **D** Pleurocystidia **E** Cheilocystidia. Scale bars: 12 µm (**A**), 20 µm (**B–E**).

Basidiospores (12.0)13.5–15.0(16.0) × (9.5)10.5–12.0(13.0) × (7.5)9.5–10.5(12.0) µm, on average 14.0 × 11.3 × 9.7 µm, Q_1_ = 1.3–1.5, Q_2_ = 1.4–1.5, avQ = 1.4; in face view mostly rounded triangular to heart shaped, rarely ovoid to subglobose, in side view ellipsoid to oblong, with eccentric germ pore of 1–1.5 µm diam, dark to blackish in KOH. Basidia 24–31 × 8–12 µm, clavate to cylindrical, 4-spored. Cheilocystidia 55–70 × 22–29 µm, clavate, broadly clavate to broadly cylindrical. Pleurocystidia 44–67 × 19–23 µm, utriform to lageniform. Pileipellis hymeniform, consisting of clavate cells, 33–38 × 17–22 µm. Clamp connections present. Sclerocystidia absent.

#### Habitat and distribution.

Solitary to scattered on humus rich loamy soil, under *Quercus incana*. So far only known from northwest Pakistan.

#### Etymology.

The prefix “*pseudo*” means similar and “*lactea*” refers to the epithet of the species (*Parasola lactea*) that this species closely resembles. This species is known so far from low to moderate altitude mountains of northwest Pakistan.

#### Additional specimens examined.

PAKISTAN, Khyber Pakhtunkhwa Province, Shangla, 1480 m alt., 9 July 2014, Sadiq Ullah SU413 (HUP SU-413).

#### Comments.

The new species belongs to Parasola section Parasola due to the absence of sclerocystida in the pileipellis. This species resembles *Parasola lactea* and is close to that species in the molecular phylograms. However, its spores are substantially larger, closer to *P. schroeteri* or *P. hercules* in size. The spores of *P. pseudolactea* are mostly rounded triangular, rarely ovoid to subglobose in face view and larger (14.0 × 11.3 × 9.7 µm), whereas those of *P. lactea* are mostly broadly ovoid to subglobose, rarely rounded triangular in face view, and comparatively smaller (10.73 × 8.81 × 6.73 μm). Other species similar to the new taxon are *P. megasperma* and *P. plicatilis*. Both these species share pileus color with *P. pseudolactea*. Lamellae of *P. megasperma* and *P. plicatilis* are separated from the stipe by a pseudocollarium, whereas in *P. pseudolactea*, a pseudocollarium is generally absent. Basidiospores are more ellipsoid rarely ovoid in face view and on average 16.5 × 10.66 × 8.5 μm in *P. megasperma*. Basidiospore shape is quite variable in *P. plicatilis*, in face view mostly limoniform-subhexagonal, rarely ovoid, in side view broadly ellipsoid, on average 12.41 × 8.21 × 7.14 μm (Nagy et al. 2010). Comparison of morpho-anatomical features of *P. pseudolactea* with regards to other species of the genus *Parasola* are set out in Table 2, where the new species can be differentiated by careful comparison of the morphology of its basidiospores.

### Parasola auricoma

Taxon classificationFungiAgaricalesPsathyrellaceae

(Pat.) Redhead, Vilgalys & Hopple, Taxon 50: 235. 2001.

Figures 6 , 8

#### Synonymy.

*Coprinus auricomus* Pat., Tab. analyt. Fung. 5: 200, 1886.

#### Description.

Pileus 15–30 mm diam, convex to broadly convex, deeply plicate towards the margin, light grayish-brown (2.5YR 5/2) to grayish reddish-brown (2.5YR 3/2); disc indistinctly umbonate to umbilicate, dark reddish orange (7.5R 4/8) to grayish reddish orange (2.5YR 5/6). Lamellae free and remote, pseudocollarium absent, closed, initially concolorous with pileus, later on dark black, finally losing turgor and collapsing. Stipe 40–65 × 2–5 mm, equal, smooth, central, hollow, without annulus.

**Figure 8.**
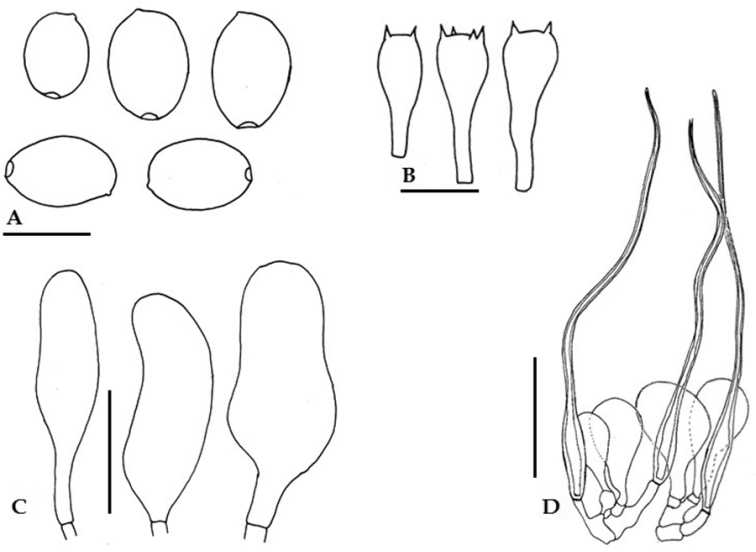
Anatomical features of *Parasola auricoma* (LAH-SHP-7). **A** Basidiospores **B** Basidia **C** Cheilocystidia **D** Pileipellis. Scale bars: **A** = 10 µm, **B–D** = 20 µm.

Basidiospores (10.5)12.5–13.5(15.0) × (8.0)8.5–9.5(10.0) × (7.0)8.0–9.0(10.0) μm, on average 12.9 × 9.0 × 8.5 μm, Q_1_ = 1.5–1.6, Q_2_ = 1.3–1.4, avQ = 1.5; in face view subcylindrical to ellipsoid or ovoid, in side view ellipsoidal to elliptical; with central germ-pore, 2–2.5 μm diam, wall 1.5 µm thick, strong reddish-brown to blackish in KOH. Basidia 30–38 × 7–11 μm, clavate to subcylindrical, 2- or 4-spored. Cheliocystidia 33–45 × 12–25 μm, subclavate to subglobose, abundant. Pleurocystidia 30–40 × 11–15 μm, cylindrical to clavate, pale brown at the base, rare. Sclerocystidia 90–170 × 4–7 μm, dark brown, with acute apex and bulbous base, wall 1.5–2 μm thick. Clamp connection present.

#### Specimens examined.

Pakistan, Khyber Pakhtunkhwa Province, Malakand, Kharkai, alt. 460 m, scattered in grassland under herbaceous plants, 10 August 2014, S. Hussain SHP6 (LAH-SHP-6), 10 August 2014, S. Hussain SHP7 (LAH-SHP-7), Malakand, Qaldara 10 August 2014, S. Hussain SHP11 (LAH-SHP-11); Khyber Pukhtunkhwa Province, Swat, Kanju Township, alt. 1023 m, 27 July 2017, S. Hussain SHP34 (SWAT SHP-34).

### Parasola lilatincta

Taxon classificationFungiAgaricalesPsathyrellaceae

(Bender & Uljé) Redhead, Vilgalys & Hopple, Taxon 50: 236. 2001.

Figures 6 , 9

#### Synonymy.

*Coprinus lilatinctus* Bender & Uljé, Persoonia 16: 373, 1997.

#### Description.

Pileus 20–30 mm diam, hemispheric to pulvinate, smooth, deeply plicate towards margin, yellow brown (2.5R 9/2–5R 9/2) to grayish red brown (2.5R 7/2–5R 7/2); disc slightly depressed, brilliant orange (2.5YR 8/12 – 5YR 8/12) to strong orange (2.5YR 6/12–5YR 6/12). Lamellae free, separated from the stipe by pseudocollarium, distant, lamellae edge blackish while faces initially concolorous with the pileus but later on black and finally losing turgor and collapsing. Stipe 40–60 × 1 mm, equal, smooth, white, fragile, without annulus with slightly sub-bulbous base.

**Figure 9.**
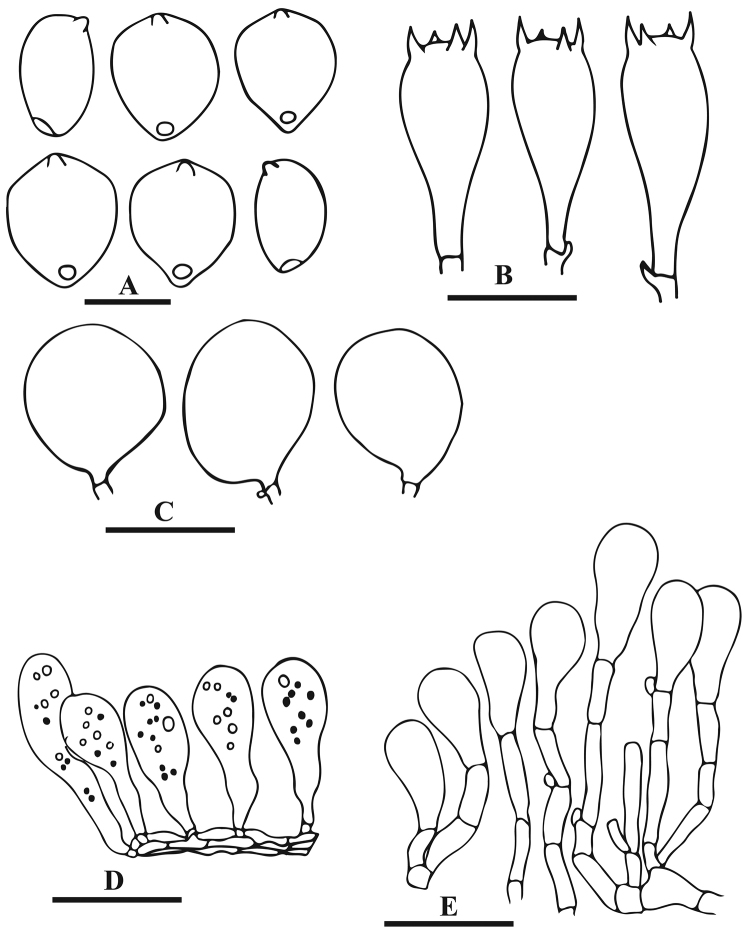
Anatomical features of *Parasola lilatincta* (LAH-SHP-8). **A** Basidiospores **B**Basidia **C** Pleurocystidia **D** Cheilocystidia **E** Pileipellis. Scale bars: **A** = 10 µm, **B–E** = 20 µm.

Basidiospores (12)13–14.5(15.5) × (11.5)12–12.5(13.5) × (6.0)8.5–11(13.5) μm, on average 14.5 × 12.5 × 9.9 μm, Q_1_ = 1.1–1.2, Q_2_ = 1.2–1.5, avQ = 1.3; in the face view rounded triangular to subglobose, in side view ovoid to amygdaliform, with eccentric germ-pore of 2–2.5 μm diam; wall upto 2 µm thick, dark brown in KOH. Basidia 17–22 × 6–9 μm, 4-spored, cylindrical to clavate, hyaline in KOH. Cheilocystidia 25–29 × 23–26 μm, rounded to globose, rare. Pleurocystidia 34–40 × 11–14 μm, cylindrical to subclavate. Pileipellis of clavate cells, 33–37 × 9–12 μm, with rounded apex, bright yellow at the base. Clamp connections present in most of the tissues. Sclerocystidia absent.

#### Specimens examined.

PAKISTAN, Khyber Pakhtunkhwa Province, Malakand, Qaldara, alt. 430 m, scattered under herbaceous plants, 11 August 2014, S. Hussain SHP-8, SHP-31, SHP-12 (LAH SHP-8; LAH SHP-31; LAH SHP-12); Khyber Pakhtunkhwa Province, Swat, Kanju Township, alt. 1023 m, on road trails, 27 July 2017, S. Hussain SHP35 (SWAT SHP-35).

## Discussion

The incorporation of molecular phylogenetics has significantly benefited the systematic and taxonomic studies of coprinoid mushrooms. These mushrooms are deliquescent or, at least, have morphological characters like gill cystidia, coloration and surface features that are quickly changed during basidioma maturation. So morphology based taxonomy of coprinoid mushrooms is always a difficult task for mushroom biologists. In the present study two new species of mushroom genus *Parasola* are described from Pakistan, based on morphological and molecular data.

On account of absence of sclerocystidia in the pileipellis, both the new species *P. glabra* and *P. pseudolactea* belong to section Parasola of genus *Parasola*. *Parasola glabra* with light gray to moderate gray pileus was collected in Malakand region of Pakistan. This region is rich in diversity of *Parasola* species (Hussain et al. 2016, 2017). The new species *P. glabra* with broadly ovoid to oblong, some with rhomboidal basdiospore is closely related to *P. hercules*. Morphological features of *P. glabra* are discussed with other species of section Parasola genus *Parasola*, set out in Table 2. Phylogenetic inference of *P. glabra* based on ITS and combined ITS-28S datasets was strongly supported (MLB 100% and BPP 1). While in combined ITS-28S-*TEF1a* dataset, *P. glabra* was represented by single specimen and was poorly recovered.

Similarly, the second new species *P. pseudolactea* in this study was collected in Shangla district, Khyber Pakhtunkhwa province of Pakistan. This species with yellow brown to dull brown pileus, basidiospores mostly rounded triangular to heart shape, was found in a *Quercus* forest. The species most closely related to *P. pseudolactea* on the basis of basidiospore morphology is *P. lactea*. Basidiospores are mostly rounded triangular to heart shape, rarely ovoid to subglobose in face view in *P. pseudolactea*; while spores are ovoid to subglobose, rarely rounded triangular in face view in *P. lactea*. A poorly described species *P. subprona* (Cleland) J.A. Simpson & Grgur. with elliptical basidiospores (15 × 8 µm) can be differentiated from both the new species on account of central germ-pore (Grgurinovic 1997). Phylogenetic analyses recovered *P. pseudolactea* well supported in ITS, combined ITS-28S and combined ITS-28S-*TEF1a* datasets (Figures 1, 2 and 3), respectively. Along with these new species, collections of *P. auricoma* and *P. lilatincta* from Pakistan were also documented in this study. The phylogenetic separation of *P. auricoma* collected in Pakistan from European collections (albeit into adjacent clades) suggests that the taxon from Pakistan may be a distinct, previously undescribed species. However, morphological features do not yet provide a basis for distinguishing separate species.

## Conclusion

It is concluded form this study that low altitude mountains of northern Khyber Pakhtunkhwa Province of Pakistan are rich in the diversity of *Parasola* and other coprinoid mushrooms.

## Supplementary Material

XML Treatment for Parasola glabra

XML Treatment for Parasola pseudolactea

XML Treatment for Parasola auricoma

XML Treatment for Parasola lilatincta
